# Comparison of the effect of combined usage of povidone-iodine irrigation and topical vancomycin powder to the use of povidone-iodine irrigation alone on the periprosthetic joint infection incidence rate in patients undergoing primary total hip and knee arthroplasty: a protocol for multicenter prospective randomized clinical trial

**DOI:** 10.1186/s13063-024-08306-3

**Published:** 2024-07-10

**Authors:** Michał Kułakowski, Karol Elster, Mateusz Szymczak, Paweł Ślęczka, Mariusz Baumgart, Aleksandra Królikowska, Paweł Reichert

**Affiliations:** 1 Orthopaedic and Trauma Surgery Department, Independent Public Healthcare Center in Rypin, Rypin, Poland; 2 Trauma and Orthopedic Surgery Department, Independent Public Healthcare Center in Myslenice, Myslenice, Poland; 3 Department of Normal Anatomy, Ludwik Rydygier Collegium Medicum in Bydgoszcz, Nicolaus Copernicus University in Toruń, Toruń, Poland; 4 Ergonomics and Biomedical Monitoring Laboratory, Department of Physiotherapy, Faculty of Health Sciences, Wroclaw Medical University, Wroclaw, Poland; 5 Department of Orthopedics, Traumatology and Hand Surgery, Faculty of Medicine, Wroclaw Medical University, Wroclaw, Poland

**Keywords:** Arthroplasty, Orthopedics, Periprosthetic joint infection, Postoperative complications, Total hip replacement, Total knee replacement

## Abstract

**Background:**

With the increasing number of joint replacement surgeries, periprosthetic joint infection (PJI) has become a significant concern in orthopedic practice, making research on PJI prevention paramount. Therefore, the study will aim to compare the effect of combined usage of povidone-iodine and topical vancomycin powder to the use of povidone-iodine alone on the PJI incidence rate in patients undergoing primary total hip (THA) and total knee arthroplasty (TKA).

**Methods:**

The prospective randomized clinical trial will be conducted in two independent voivodeship hospitals with extensive experience in lower limb arthroplasties. The studied material will comprise 840 patients referred to hospitals for primary THA or TKA. The patients will be randomly allocated to two equal groups, receiving two different interventions during joint replacement. In group I, povidone-iodine irrigation and consecutively topical vancomycin powder will be used before wound closure. In group II, only povidone-iodine lavage irrigation will be used before wound closure. The primary outcome will be the incidence rate of PJI based on the number of patients with PJI occurrence within 90 days after arthroplasty. The occurrence will be determined using a combined approach, including reviewing hospital records for readmissions and follow-up phone interviews with patients. The infection will be diagnosed based on Musculoskeletal Infection Society criteria. The chi-square test will be used to compare the infection rates between the two studied groups. Risk and odds ratios for the between-groups comparison purposes will also be estimated. Medical cost analysis will also be performed.

**Discussion:**

A randomized clinical trial comparing the effect of combined usage of povidone-iodine irrigation and vancomycin powder to the use of povidone-iodine irrigation alone in preventing PJIs after primary arthroplasty is crucial to advancing knowledge in orthopedic surgery, improving patient outcomes, and guiding evidence-based clinical practices.

**Trial registration:**

ClinicalTrials.gov NCT05972603. Registered on 2 August 2023.

## Administrative information

Note: the numbers in curly brackets in this protocol refer to SPIRIT checklist item numbers. The order of the items has been modified to group similar items

**Table:** 

Title {1}	Comparison of the effect of combined usage of povidone-iodine irrigation and topical vancomycin powder to the use of povidone-iodine irrigation alone on the periprosthetic joint infection incidence rate in patients undergoing primary total hip and knee arthroplasty: a protocol for multicenter prospective randomized clinical trial
Trial registration {2a and 2b}	ClinicalTrials.gov Identifier: NCT05972603. Registered 2 August 2023, https://classic.clinicaltrials.gov/ct2/show/NCT05972603
Protocol version {3}	2 August 2023 Identifier: NCT05972603
Funding {4}	The study will get no external funding.
Author details {5a}	Michał Kułakowski^1^, Karol Elster^1^, Mateusz Szymczak^1^, Paweł Ślęczka^2^, Mariusz Baumgart^3^, Aleksandra Królikowska^4^, Paweł Reichert^5^ ^1^Orthopaedic and Trauma Surgery Department, Independent Public Healthcare Center in Rypin, Rypin, Poland ^2^Trauma and Orthopedic Surgery Department, Independent Public Healthcare Center in Myslenice, Myslenice, Poland ^3^Department of Normal Anatomy, Ludwik Rydygier Collegium Medicum in Bydgoszcz, Nicolaus Copernicus University in Toruń, Poland ^4^Ergonomics and Biomedical Monitoring Laboratory, Department of Physiotherapy, Faculty of Health Sciences, Wroclaw Medical University, Wroclaw, Poland ^5^Department of Orthopedics, Traumatology and Hand Surgery, Faculty of Medicine, Wroclaw Medical University, Wroclaw, Poland
Name and contact information for the trial sponsor {5b}	Not applicable as the study will get no external funding.
Role of sponsor {5c}	Not applicable as the study will get no external funding.

## Introduction

### Background and rationale {6a}

Periprosthetic joint infections (PJIs) are among the most severe complications following total knee (TKA) and total hip (THA) arthroplasties [1, 2]. With the increasing number of joint replacement surgeries, PJIs have become a significant concern in orthopedic practice [3]. Therefore, research focused on preventing these infections is of paramount importance [4].

A commonly employed prophylactic measure to prevent infections is povidone-iodine, which has been used as a preoperative skin antiseptic to reduce the risk of surgical site infections [5, 6]. Irrigating the joint space with povidone-iodine is an additional measure to prevent PJI [7, 8]. Povidone-iodine is generally cost-effective and readily available. Incorporating it into the surgical protocol as an irrigation agent represents a practical and economically viable strategy to enhance PJI prevention efforts in TKA and THA patients [9]. Still, there exists a need to confirm the effectiveness of its usage due to controversial results and a limited number of high-level evidence studies [7, 9–12].

Vancomycin is a potent antibiotic effective against a wide range of bacteria, including methicillin-resistant *Staphylococcus aureus* (MRSA) and other drug-resistant strains [13]. Its topical application as a targeted approach to prevent bacterial colonization around the surgical site has been widely studied [14, 15]. The literature on the efficacy of vancomycin as a preventive method for PJI in TKA and THA patients shows notable disagreement [16]. While specific studies support its use, others do not, primarily due to the scarcity of prospective randomized clinical trials (RCT) in this domain [17–20].

The question remains whether additional topical use of vancomycin powder between povidone-iodine irrigation and wound closure would be more beneficial in preventing PJI than using povidone-iodine irrigation alone. As surgeons and healthcare providers need reliable evidence to make informed decisions about perioperative interventions, conducting an RCT ensures a high level of scientific validity, providing robust evidence on the efficacy of different prophylactic strategies against PJI. Well-designed RCTs give the necessary data to guide clinical practice, ensuring patients receive the best care based on scientific evidence [21].

## Objectives {7}

The study compares the effect of combined usage of povidone-iodine irrigation and topical vancomycin powder to povidone-iodine irrigation alone on the periprosthetic joint infection incidence rate in patients undergoing primary THA and TKA. It is hypothesized that the periprosthetic joint infection incidence rate is significantly lower in patients undergoing total hip and knee arthroplasty with combined povidone-iodine irrigation and topical vancomycin powder compared to those with povidone-iodine irrigation alone.

## Trial design {8}

The trial will have a parallel-group, two arms, and a superiority design with an allocation ratio 1:1.

## Methods: participants, interventions, and outcomes

### Study setting {9}

For the present study purposes, will be included patients referred for surgeries to departments in two independent voivodeship hospitals, namely the Orthopaedic and Trauma Surgery Department, Independent Public Healthcare Center in Rypin, Rypin, Poland, and the Trauma and Orthopedic Surgery Department, Independent Public Healthcare Center in Myslenice, Myslenice, Poland, constituting the settings where the data will be collected. The three other study settings, precisely the Department of Normal Anatomy, Ludwik Rydygier Collegium Medicum in Bydgoszcz, Nicolaus Copernicus University in Toruń, Poland, Ergonomics and Biomedical Monitoring Laboratory, Department of Physiotherapy, Faculty of Health Sciences, Wroclaw Medical University, Wroclaw, Poland, and the Department of Orthopedics, Traumatology and Hand Surgery, Faculty of Medicine, Wroclaw Medical University, Wroclaw, Poland, will be involved in conducting the trial. Still, no data will be collected at those three units.

### Eligibility criteria {10}

The studied sample will include the first 840 patients who meet all the inclusion criteria and do not meet any exclusion criteria.

Inclusion criteria: patients undergoing primary THA or TKA at one of the two participating settings where the data will be collected due to osteoarthritis or rheumatoid arthritis; patients aged 18 years or older; willingness to provide informed consent for participation in the study.

Exclusion criteria: emergency cases requiring immediate surgery due to trauma or acute conditions; known allergy or sensitivity to iodine or vancomycin; history of previous joint infection in the affected joint; immunocompromised individuals, including those with active cancer under treatment, organ transplantation, or chronic immunosuppressive therapy; patients with chronic systemic infections, such as active tuberculosis or chronic osteomyelitis in a different joint; patients undergoing revision hip or knee arthroplasty rather than primary procedures; participation in another clinical trial with interventions that may confound the outcomes of the current study; severe medical comorbidities (e.g., severe cardiovascular disease, end-stage renal disease) that could significantly impact the patient’s ability to undergo surgery or follow the study protocol; inability or unwillingness to comply with the study follow-up schedule; known bacterial resistance to vancomycin based on preoperative cultures; usage of any antibiotics within the first 90 days postoperatively that are not included in the study protocol.

The departments in two independent voivodeship hospitals, namely the Orthopaedic and Trauma Surgery Department, Independent Public Healthcare Center in Rypin, Rypin, Poland, and the Trauma and Orthopedic Surgery Department, Independent Public Healthcare Center in Myslenice, Myslenice, Poland, constituting the settings where the data will be collected set up the study based on their experience in lower limb arthroplasties and previous scientific cooperation. Also, the individuals performing the interventions were chosen based on their clinical and scientific expertise.

### Who will take informed consent? {26a}

In the two trial settings, a determined person will be responsible for informing participants about the aim of the study and the approach to be used and gaining their signed consent for participation (KE and MB). The same two study coordinators will be responsible for achieving informed consent from participants for using exemplary intraoperative or postoperative photos to disseminate the trial, i.e., publishing an article or utilizing the picture at scientific conferences. The images will be taken, so it will be impossible to identify the person in the photo.

### Additional consent provisions for collection and use of participant data and biological specimens {26b}

On the consent form, participants will be asked if they agree to use their data should they choose to withdraw from the trial. Participants will also be asked for permission to allow the research team from departments in two independent voivodeship hospitals to share relevant data with the researchers from the three participating universities or with regulatory authorities, where applicable. This trial does not involve collecting biological specimens for storage.

## Interventions

### Explanation for the choice of comparators {6b}

The combined use of betadine irrigation and vancomycin powder will be compared against the usage of betadine irrigation only, which can be considered a standard form of treatment.

### Intervention description {11a}

The studied interventions will be conducted intra-operatively, precisely during THA or TKA. In group I, before wound closure, both povidone-iodine irrigation and topical vancomycin powder will be applied. In group II, only povidone-iodine irrigation will be provided before wound closure.

#### Povidone-iodine irrigation

For the study purposes, Braunol® (Braun Melsungen AG, 34209 Melsungen, Germany) povidone-iodine-solution will be used. To create the dilute solution, the scrub nurse draws up 17.5 mL of 10% povidone-iodine with a syringe and mixes it with 500 mL of sterile isotonic sodium chloride solution. This results in a dilution of 0.35% povidone-iodine for use before wound closure. After implantation of the prosthetic components, the wound is soaked with 500 mL of the povidone-iodine solution for 3 min, followed by pulsatile lavage with 1 L of isotonic sodium chloride solution without antibiotics. Before final closure, Kodan (45 g 2-propanolum + 10 g 1-propanolum + 0.2 g 2-biphenylol/100 g; Schulke & Mayr GmbH, Germany) is applied to the skin surrounding the incision [8].

In group I, vancomycin powder will be used consecutively. In the group II standard, wound closure will be performed.

#### Topical vancomycin powder application

A dosage of 1.0 g of vancomycin hydrochloride powder will be administrated (Edicin®, Sandoz, GmbH, 6250 Kundl, Austria). The vancomycin powder is distributed evenly over the prepared surgical site, focusing on the bone surfaces, soft tissues, and surrounding areas. Care is taken to avoid direct contact of the vancomycin powder with the prosthetic components to prevent any potential issues with implant integration. No suction is used. Consecutively standard wound closure is proceeded.

### Criteria for discontinuing or modifying allocated interventions {11b}

Criteria for discontinuing allocated interventions for a given trial participant: voluntary withdrawal or request for discontinuation by the participant due to any reason; diagnosis of an unanticipated medical condition that contraindicates the continuation of the allocated intervention; confirmation of pregnancy during the study period; persistent non-compliance with the study protocol or inability to follow the assigned intervention regimen; concomitant care and interventions prohibited during the trial stated in details in {11d}.

### Strategies to improve adherence to interventions {11c}

The strategy to improve adherence to intervention protocols will include:

- Development of clear and comprehensive protocols outlining the procedures for both intervention arms.

Step-by-step instructions for applying povidone-iodine irrigation and topical vancomycin powder will be provided. It will be ensured that the two settings responsible for data collection receive and understand the standardized protocol.

b) Conduction of training sessions for the surgical teams at each participating center to ensure a consistent understanding of the interventions.

Education on the rationale behind the study will be provided, emphasizing the importance of adherence for accurate results.

c) Implement a system for regular monitoring and site audits to assess adherence to the study protocols. This will include on-site visits to observe and confirm compliance with intervention procedures.
d) Establishing effective communication channels, such as email updates, to keep all study sites informed about any protocol modifications, clarifications, or important updates related to the interventions.
e) Providing regular feedback to participating sites regarding their adherence to the intervention protocols.

This can include performance reports highlighting areas of excellence and areas needing improvement.

f) Education of participants concerning the importance of remaining vigilant and informing their attending physician from the setting where they were operated on if they experience any disturbing symptoms.

### Relevant concomitant care permitted or prohibited during the trial {11d}

#### Permitted concomitant care and interventions

- Standard of care

Participants will receive standard-of-care interventions considered routine and necessary for their condition. This includes standard perioperative care, pain management, and rehabilitation practices.

b) Routine analgesics and anticoagulant intake

Perioperative usage of analgesics and anticoagulants that align with standard clinical practice and guidelines is permitted. During the postoperative period, analgesics (morphine and ketoprofen) will be used intravenously. Also, anticoagulant prophylaxis will be administered, and enoxaparin, precisely Clexane® 4000 U/0.4 mL (40 mg), will be used once a day.

c) Postoperative wound care

Standard postoperative wound care practices, such as sterile dressing changes, are allowed.

d) Physical therapy

Participants will undergo physical therapy as part of routine postoperative care.

e) Management of complications

Interventions required to manage unexpected complications or adverse events are permitted. However, if any unexpected complications or adverse events occur, they will be documented and reported along with the study results.

f) Routine follow-up care

Routine follow-up visits and assessments are part of standard postoperative care.

#### Prohibited concomitant care and interventions

- Use of additional antimicrobial agents

Participants are prohibited from using additional antimicrobial agents (other than those specified in the study protocol) during the trial period. This restriction helps ensure that other antibiotics do not confound the effects of the studied interventions.

b) Additional surgical interventions

Any additional surgical interventions related to the joint being studied (hip or knee arthroplasty) during the trial period are prohibited. This also includes other procedures that could affect the risk of periprosthetic joint infection.

c) Use of alternative topical agents

Participants are restricted from using alternative topical agents or antiseptics on the surgical site that are not part of the study interventions. This ensures that using other substances does not compromise the impact of the studied interventions.

d) Changes in standard surgical practices

Modifications to standard surgical practices or infection prevention protocols are prohibited during the trial period. This is to maintain consistency in the surgical procedures across all participants.

e) Participation in other clinical trials

Participants are prohibited from participating in other clinical trials with interventions that could impact the outcomes being studied in the present trial. This is to avoid potential interactions between interventions from different studies.

f) Use of antibiotics beyond protocol guidelines

Participants are restricted from using antibiotics beyond what is specified in the study protocol.

g) Use of investigational drugs or devices

Participants are prohibited from using investigational drugs or devices that are not a part of the present study protocol. This ensures that the potential effects of these interventions are not confounded with the primary study interventions.

h) Use of alternative infection prevention measures

Participants are restricted from using alternative infection prevention measures or procedures that are not part of the present study protocol. This includes any additional measures that could influence the risk of periprosthetic joint infection.

i) Significant changes in rehabilitation protocols

Significant changes in rehabilitation protocols during the trial period are prohibited. This ensures consistency in postoperative care and minimizes confounding factors related to rehabilitation practices.

### Provisions for post-trial care {30}

No provisions for ancillary and post-trial care and compensation to those who suffer harm from trial participation are assumed apart from those covered by standard hospital insurance.

### Outcomes {12}

The primary outcome will be a PJI incidence, expressed as an incidence rate. The incidence rate will be based on the occurrence of PJI in studied patients within 90 days after arthroplasty. All suspected PJIs will be confirmed by manual chart review using the Musculoskeletal Infection Society (MSIS) criteria for diagnosing PJI.

Any adverse events and other unintended effects of trial interventions or trial conduct within 90 days after arthroplasty will be documented.

#### Baseline data

Comprehensive baseline data will be collected, including age, gender, body mass index (BMI), presence of diagnosed hyper-pressure, presence of diagnosed metabolic syndrome, and class according to the American Society of Anesthesiologists classification (ASA class).

#### Efficacy outcomes

The primary efficacy outcome, the PJI rate, directly addresses the central question of the study. It is clinically relevant as it quantifies the occurrence of a critical complication in patients undergoing THA and TKA.

#### Harm outcomes

Documenting adverse events and other unintended effects related to interventions is critical for understanding potential harms. This information informs clinicians about the safety profile of the preventive measures, ensuring that the benefits outweigh the risks.

#### Medical cost analysis

Medical cost analysis is important in terms of evaluating the economic impact of combined povidone-iodine irrigation and topical vancomycin powder versus povidone-iodine irrigation alone on the incidence of PJI in patients undergoing THA and TKA.

### Participant timeline {13}

See Table 1.

**Table 1:** A schematic diagram of the schedule of enrolment, interventions, assessments, and visits for participants

	**Study period**					
	**Enrolment**	**Allocation**	**Post-allocation**			**Close-out**
**Timepoint**	**−t**_**1**_	**0**	**t**_**1**_	**t**_**2**_	**t**_**3**_	**t**_**4**_
**Enrolment**						
**Eligibility screen**	X					
**Informed consent**	X					
**Allocation**		X				
**Interventions**						
**Povidone-iodine irrigation and topical vancomycin powder**			X			
**Povidone-iodine irrigation**			X			
**Assessments**						
**Baseline variables** **age, gender, BMI, presence of hyper-pressure and metabolic syndrome, ASA class**	X	X				
**Occurrence of PJI**				X	X	X
**Occurrence of adverse events and harms**				X	X	X
**Medical cost analysis**						X

−t_1_, between admission to the hospital and TKA or THA; t_1_, TKA or THA; t_2_, follow-up visits 2 weeks postoperatively; t_3_, follow-up visits 2 weeks postoperatively; t_4_, phone contact at 90 days postoperatively

### Sample size {14}

The minimal needed sample was calculated using G*Power based on previous studies by Gundtoft (2015) and Buchalter (2021) with 1 − ß of 0.80, α = 0.05, and exceeded 762 [11, 22]. Considering the risk of withdrawal, the studied sample was determined to comprise 840 participants divided into two equal groups of 420 participants.

### Recruitment {15}

For the present study purposes, there will be recruited patients admitted to one of the two study sites to undergo THA or TKA. To ensure that an adequate number of participants will be recruited, two settings with extensive experience in lower limb arthroplasties were involved. In the Orthopaedic and Trauma Surgery Department in the Independent Public Healthcare Center in Rypin, Rypin, Poland, there are performed annually, on average, 300 lower limb arthroplasties, including 300 THA and 200 TKA. The employees of the 20-bed department include seven specialists in orthopedics and traumatology with many years of experience performing THA and TKA. In the Trauma and Orthopedic Surgery Department in the Independent Public Healthcare Center in Myslenice, Myslenice, Poland, there are an average of 400 lower limb arthroplasties per year, including 250 THA and 150 TKA. The employees of the 20-bed department include five specialists in orthopedics and traumatology with many years of experience in performing THA and TKA.

## Assignment of interventions: allocation

### Sequence generation {16a}

Generating the allocation sequence will be based on computer-generated random numbers. Randomization software will be employed to generate a random sequence of numbers, which are then assigned to treatment groups.

### Concealment mechanism {16b}

To implement the allocation sequence, an equal number of randomly selected sealed envelopes will be distributed to each study center. Each envelope will be sequentially numbered and correspond to a specific trial participant.

### Implementation {16c}

A designated individual external to the clinical trial team will generate the random allocation sequence using randomization software. Designated personnel at each study center will be responsible for enrolling eligible participants by assessing their eligibility, obtaining informed consent, and collecting baseline data. Study coordinators at each study center (KE and MB) will open the sequentially numbered, opaque, sealed envelopes and assign participants to the respective intervention groups based on the randomization sequence.

## Assignment of interventions: blinding

### Who will be blinded {17a}

After the assignment to interventions, trial participants and outcome assessors will be blinded. Precisely, participants will be unaware of their assigned intervention. Achieving blinding for outcome assessors will involve using independent assessors who do not have access to information about the participants’ interventions. Standardized assessment protocols will be used to reduce potential bias. Additionally, the dataset provided to the analysts will be masked, meaning that information about intervention groups will be replaced with codes. Analysts will perform the analysis without knowing the interventions corresponding to the codes.

### Procedure for unblinding if needed {17b}

Unblinding is permissible in a medical emergency, where knowledge of the participant’s treatment assignment is essential for appropriate and timely medical management. It will also be permitted in the event of a serious adverse event. Unblinding will also be conducted at the participant’s request or the request of any regulatory agencies for safety monitoring purposes or in response to regulatory queries.

## Data collection and management

### Plans for assessment and collection of outcomes {18a}

#### Baseline data

Baseline data, including age (years), gender (male/female), BMI (kg*m^−2^), presence of diagnosed hyper-pressure (yes/no), presence of diagnosed metabolic syndrome (yes/no), and ASA class (I–IV), will be collected via manual chart review. Gender, presence of diagnosed hyper-pressure, presence of diagnosed metabolic syndrome, and ASA class will be reported as a percentage of a given group of patients. The arithmetic mean and standard deviation for age and BMI will be calculated separately for each group. A 95% confidence interval (CI) will be calculated if needed.

#### Efficacy outcomes

The occurrence of PJI within 90 days after arthroplasty (yes/no) will be the incidence rate expressed as a percentage of a given group of patients. The incidence rate will be determined using a combined approach, including scheduled follow-up visits, reviewing hospital records for readmissions, and follow-up phone interviews with patients.

Scheduled follow-up visits will be conducted at predetermined intervals for each participant, precisely 2 weeks and 2 months postoperatively. The visits will be used to systematically inquire about any changes in health, new symptoms, or experiences since the previous visit. The hospital records will be analyzed for readmissions related to PJI occurrence. This approach will provide data on participants who returned to the study setting for further treatment. Medical records will be reviewed regularly every quarter. Patients will be contacted by phone 90 days postoperatively for follow-up interviews. This method will allow the investigators to capture data on patients who sought medical attention outside the study setting.

#### Harm outcomes

Any adverse events (apart from PJI) and other unintended effects of trial interventions or trial conduct that occur within 90 days after arthroplasty will be documented. Their occurrence (yes/no) will be reported as the incidence rate expressed as a percentage of a given group of patients. Also, their characteristic will be provided.

#### Medical cost analysis

The cost analysis will be conducted from the healthcare provider’s perspective, focusing exclusively on direct medical costs. Non-medical costs and indirect costs will not be included in this analysis.

The following direct medical cost categories will be included in the analysis:

- Primary surgery:
  Intervention costs, precisely costs of povidone-iodine and vancomycin powder, including acquisition, preparation, and administration.
  Procedure costs are costs associated with surgical procedures.
  Implant costs.
  Hospitalization costs are the costs of hospital stays.
  Follow-up costs include post-operative care costs, follow-up visits, and any additional treatments required.
- Complication costs are costs associated with treating periprosthetic joint infections or other complications.

Cost data will be collected for each category as follows:

- Intervention costs: cost per dose of povidone-iodine and vancomycin powder
- Procedure costs: cost per one procedure
- Cost of implant: cost per one procedure
- Hospitalization costs: cost per day of hospitalization
- Follow-up costs: cost per follow-up visit or additional treatment
- Complication costs: cost per episode of treating periprosthetic joint infections

The total costs for each patient and the average cost per patient for each group will be calculated.

The costs will be collected in Polish Zloty (PLN) and subsequently converted to United States dollars (USD) using the exchange rate on the date of the statistical analysis. The date of conversion will be reported along with the trial results.

There will be detailed instructions on completing prepared manual chart forms, ensuring consistency across data collection settings.

### Plans to promote participant retention and complete follow-up {18b}

Transparent communication will be established with participants from the outset, clearly explaining the importance of their participation and follow-up assessments. The participants will be educated on the study objectives, procedures, and the significance of their continued involvement. A thorough informed consent process emphasizing the participant’s commitment to the study and the value of their contribution to advancing medical knowledge will be ensured. Participants will be offered flexible scheduling options for follow-up assessments. In each study setting, dedicated staff will be assigned to maintain regular contact with participants, addressing any concerns or questions they may have throughout the study.

Details will be collected on the reasons for participant discontinuation, including any adverse events, personal reasons, or other factors influencing their decision.

### Data management {19}

At each setting where the data will be collected, one person will be dedicated to entering data. The data will be entered continuously. A coding system for variables to ensure consistency and facilitate data analysis will be used. The coding conventions used for different data types will be documented.

Robust data security measures will be implemented to protect participant confidentiality and comply with data protection regulations. The storage infrastructure will include both electronic and physical data. At each data collection setting, there will be specified locations where data will be stored, considering backups. Access controls to restrict data access to authorized personnel will be established. A routine schedule for data backups to prevent data loss will be developed.

A detailed data management plan includes all aspects of data management procedures, which the Principal Investigator, PI (MK), can provide upon reasonable request.

### Confidentiality {27}

Strict adherence to relevant data protection laws and regulations governing the handling of personal information will be ensured. The investigators will stay informed about updates to privacy regulations and adjust procedures accordingly.

- Informed consent process

Obtaining informed consent will be prioritized before collecting any personal information from participants. The purpose of data collection, how their information will be used, and the measures to protect confidentiality will be communicated.

b) Unique participant identifiers

Unique participant identifiers will be assigned to replace personally identifiable information in the dataset, enhancing confidentiality during data collection and analysis.

c) Limited data collection

Only essential personal information necessary for the study will be collected. Collecting sensitive data that is not directly relevant to the research objectives will be minimalized.

d) Secure storage and access controls

Data access will be restricted to authorized staff, and the roles and responsibilities of individuals handling personal data will be clearly defined.

e) Data encryption

Electronic data will be encrypted during transmission and storage to prevent unauthorized access.

f) Limited data sharing

During the trial, only the PI (MK), two study coordinators (KE and MB), and one co-investigator dedicated to auditing the trial (PR) will have access to the trial dataset, including the personal data of participants and their intervention assignment. The other co-investigators (PŚ and MS), involving the person responsible for statistical analysis (AK), will have access to the trial dataset in an anonymized form and without information concerning which group was assigned to which intervention.

After finalizing the trial, the anonymized dataset will be available in a public, open-access repository. The trial dataset, including personal information, will be appropriately stored to ensure compliance with data protection laws in each trial setting where the data will be collected. Only one person in each setting will have access to the data (MK and PŚ).

g) Secure communication channels

Transmitting sensitive data through unsecured methods such as regular email will be avoided. Secure communication channels will be used for discussions or participant information exchanges.

h) Data anonymization

Whenever possible, the data will be anonymized to further protect participant confidentiality. Before data analysis, directly identifiable information will be removed or appropriately replaced.

i) Monitoring and auditing

One co-investigator (PR) will monitor and audit data access and usage quarterly to detect and address unauthorized activities. Therefore, he will not be directly involved in the primary data collection.

j) Post-trial confidentiality:

Personal information will be retained for the time obligated by relevant data protection laws and regulations. After the retention period expires, the data will be securely archived following the obligatory rules in the setting where the data will be collected.

### Plans for collection, laboratory evaluation, and storage of biological specimens for genetic or molecular analysis in this trial/future use {33}

Not applicable. There are no plans for the collection, laboratory evaluation, and storage of biological specimens for genetic or molecular analysis in the current trial and future use in ancillary studies.

## Statistical methods

### Statistical methods for primary and secondary outcomes {20a}

SPSS Statistics version 28.0.1.0 (IBM® SPSS® Statistics, Armonk, NY, USA) and Microsoft Office Excel 365 (Microsoft Corporation, Redmond, WA, USA) will used for statistical analysis.

The primary outcome, the PJI incidence rates, will be compared between the two studied groups with the use of the chi-square test. Relative risk (RR) or risk ratio and odds ratio (OR) will also be calculated.

The between-group comparison of adverse events (apart from PJI) and other unintended effects of trial interventions or trial conduct incidence rate will be performed using a chi-square test. The chi-square test will also be used for between-group comparison of baseline categorical data expressed as a percentage of a given group of patients, including gender, presence of diagnosed hyper-pressure, presence of diagnosed metabolic syndrome, and ASA class.

For numerical data between-group comparison purposes, the Kolmogorov-Smirnov test for normality distribution will first be carried out for age, BMI, and medical cost analysis-related parameters. Consecutively, a parametric *t*-test or non-parametric Mann-Whitney *U* test for independent samples will be used.

The statistical significance was set at *p* < 0.050.

### Interim analyses {21b}

Only the PI (MK), a co-investigator (PR), and study coordinators (KE, MB) can access interim results. However, there are no plans for interim analyses regarding primary outcomes. Every quarter, PI (MK) will perform an adverse event analysis. Based on this analysis, the PI (MK) will decide on the continuation, modification, or termination of the trial. Only the PI (MK) will decide to terminate the trial.

### Methods for additional analyses (e.g., subgroup analyses) {20b}

Subgroup analyses will involve as described below.

- Joint type (hip vs. knee)

Separate analyses will be conducted for THA and TKA to identify any variations in infection rates and medical costs between these joint procedures.

b) Age groups

Participants will be stratified into different age groups to assess whether age influences infection incidence.

c) Comorbidity burden

Infection rates will be evaluated in subgroups based on the presence of diagnosed hyper-pressure, diagnosed metabolic syndrome, and severity of the ASA class.

d) BMI categories

Patients will be stratified based on BMI categories to explore the impact of obesity on infection rates.

e) Gender analysis

Separate analyses will be conducted for male and female patients to explore potential gender-related differences in infection rates.

### Methods in analysis to handle protocol non-adherence and any statistical methods to handle missing data {20c}

Protocol non-adherent participants are recognized as those who deviated from the study protocol regarding treatment procedures, medication adherence, follow-up visits, or other relevant aspects. Any non-adherence will be reported in study publications, including the frequency, types, and reasons for non-adherence. The missing data will be addressed by complete-case analyses, where participants with missing data are excluded.

### Plans to give access to the full protocol, participant-level data, and statistical code {31c}

The full study protocol can be accessed from the PI (MK) upon reasonable request. After finalizing the trial, the anonymized participant-level dataset and statistical code will be available in a public, open-access repository. The needed details on how to access the data, like the name of the repository, will be reported along with reporting of the trial results.

## Oversight and monitoring

### Composition of the coordinating center and trial steering committee {5d}

- Trial Steering Committee

The Trial Steering Committee comprises a Principal Investigator (MK) and one co-investigator (PR). Its roles involve study oversight, protocol review and approval, protocol amendments, participant safety, data and safety monitoring, quality control and assurance, recruitment and retention, communication and collaboration, ethical considerations, study dissemination, decision-making authority, and regulatory compliance. Because the trial is considered small, the PI also serves as the Project Manager.

b) Project Management Group

The Project Management Group includes key trial personnel, specifically the PI (MK), study coordinators (KE and MB), site investigators (MS and PŚ), and a data manager (AK). It is responsible for the day-to-day management and operational aspects of the trial. It handles the practical and logistical aspects, including participant recruitment, data collection, site management, and adherence to the trial protocol.

c) Coordinating Center

The Coordinating Center will involve PI (MK), study coordinators (KE and MB), site investigators (MS and PŚ), and a data manager (AK). At each study center, there will be one study coordinator (KE and MB) whose responsibilities involve protocol implementation, regulatory compliance, patient recruitment, and informed consent, data collection and management, communication and collaboration, study monitoring, adverse event reporting, logistical coordination, participant follow-up, problem resolution, study closeout, documentation, and record keeping. The two coordinators will be in continuous contact and will meet when needed. Site investigators (MS and PŚ) will be responsible for surgeries and will cooperate with hospital employees on a daily basis.

### Composition of the data monitoring committee, its role and reporting structure {21a}

The PI (MK), one co-investigator (PR), and study coordinators (KE and MB) compose a data monitoring committee whose responsibilities involve safety oversight, interim data review, efficacy monitoring, data quality and integrity, unblinding procedures, communication with investigators, confidentiality, and ethical considerations.

### Adverse event reporting and harms {22}

Plans for collecting, assessing, reporting, and management of solicited and spontaneously reported adverse events and other unintended effects of trial interventions or trial conduct:

1. Definitions, characteristics, and examples of adverse events and other unintended effects
  1.1. Adverse event (AE)

Definition: An adverse event (AE) is any undesirable and unintended medical occurrence or finding that happens during the course of the study, whether or not it is considered related to the trial interventions. AEs can encompass various events, including symptoms, signs, illnesses, or abnormal laboratory findings.

Characteristics: AEs may be expected, reflecting known risks associated with the surgical procedures or interventions. AEs may also be unexpected, representing unforeseen complications or outcomes.

Examples: Mild surgical site pain following arthroplasty or temporary local skin irritation after applying povidone-iodine or vancomycin powder.

1.2. Serious adverse event (SAE)

Definition: A serious adverse event (SAE) is any adverse event that results in death, is life-threatening, requires inpatient hospitalization or prolongation of existing hospitalization, results in persistent or significant disability or incapacity, or requires medical or surgical intervention to prevent one of the above outcomes.

Characteristics: SAEs are events of a more severe nature that can pose a threat to the participant’s life, health, or well-being.

Examples: Severe allergic reaction requiring immediate medical intervention. Postoperative infection, the primary outcome in the present study, is also an example of an SAE.

Other unintended effects

Definition: Other unintended effects refer to any unexpected, adverse events, outcomes, or consequences related to the trial interventions or trial conduct that do not meet the criteria for classification as an AE or SAE. These effects may impact participant safety, well-being, or the overall conduct of the study.

Characteristics: Other unintended effects may include unexpected challenges, inconveniences, or complications that do not meet the severity criteria of AEs or SAEs but are still relevant for evaluation. They are events that were not anticipated but are not severe enough to be classified as AEs or SAEs.

Examples: Unexpected technical difficulties during intervention administration or minor, transient side effects that do not meet the criteria for AEs.

2. Collection of adverse events and other unintended effects
  2.1. Identification of adverse events and other unintended effects

A systematic process for identifying adverse events throughout the study period will be established. The study personnel will actively monitor and document any signs or symptoms that could indicate an adverse event.

2.2. Scheduled follow-up visits

Follow-up visits will be scheduled at predetermined intervals for each participant, precisely 2 weeks and 2 months postoperatively. The visits will be used to systematically inquire about any changes in health, new symptoms, or experiences since the previous visit.

2.3. Participants interviews

During each follow-up visit, thorough participant interviews will be conducted using standardized questionnaires. Questions and prompts will also be asked to encourage participants to report any perceived changes or issues related to the interventions.

2.4. Participant education

The participants will be educated about the importance of reporting any changes in their health, even if they believe it is unrelated to the study interventions. They will receive clear instructions on communicating adverse events between scheduled visits.

2.5. Medical records

The medical records of participants will be utilized as valuable sources of information. The records will be regularly reviewed for documented events, diagnoses, or treatments related to the study.

2.6. Continuous monitoring

A system for continuous monitoring of participant health status will be established, so apart from scheduled visits, the participants will be contacted by phone 90 days postoperatively. The study personnel will be encouraged to promptly address and document any participant concerns, even between scheduled visits.

3. Sources of information and frequency of data collection
  3.1. Participants interviews

Frequency: Participant interviews will be conducted at each scheduled follow-up visit and by phone 90 days postoperatively in case there is no prior information concerning the occurrence of periprosthetic joint infection.

Source: Primary source of information directly from the participant.

3.2. Medical records review

Frequency: Medical records will be reviewed regularly every quarter.

Source: Secondary source of information from healthcare providers.

3.3. Continuous monitoring

Frequency: Ongoing, continuous monitoring.

Source: Continuous observation and proactive communication with participants and healthcare providers.

3.4. Participant education

Frequency: Ongoing education will be provided throughout the study.

Source: Education materials and interactions designed to empower participants to report changes promptly.

4. Reporting timelines

Adverse events that are non-serious and expected should be reported during scheduled data collection visits. Serious and unexpected adverse events require expedited reporting to the PI (MK) within 24 to 72 h of awareness or as per regulatory requirements.

5. Reporting responsibilities
  5.1. Principal Investigator (MK)
    Assuming ultimate responsibility for the study’s conduct, including adverse event reporting
    Reviewing and assessing the severity, relatedness, and significance of reported adverse events
    Making decisions on the continuation, modification, or termination of the study based on adverse event assessments
    Ensuring accurate reporting of adverse events to relevant parties, including the ethics committee and regulatory authorities
  5.2. Study coordinators (KE and MB)
    Serving as the primary point of contact for participants regarding adverse events
    Collecting and documenting information on adverse events during participant visits
    Ensuring completeness and accuracy of adverse event reports in the case report forms
    Coordinating with site investigators to collect additional information or follow-up data on adverse events
    Facilitating the timely submission of adverse event reports to the PI (MK), ethics committee, and regulatory authorities
  5.3. Site investigators (MS and PŚ)
    Monitoring participants for adverse events throughout the study period
    Promptly documenting adverse events in the case report forms during participant visits
    Investigating and collecting additional information related to adverse events as needed
    Collaborating with the study coordinator to ensure accurate and timely reporting of adverse events
    Notifying the PI (MK) and study coordinator immediately of serious or unexpected adverse events
  5.4. Data manager (AK)
    Ensuring that adverse event data are accurately entered into the study database
    Conducting regular reviews of adverse event documentation to identify discrepancies or missing information
    Working collaboratively with the study coordinator to address any data-related issues promptly

6. Documentation and record-keeping

So-called case report forms will be used to document adverse events and other unintended effects. The forms will be stored with the rest of the trial documentation.

7. Management of adverse events

Upon identification of an adverse event, an immediate assessment of its severity will be conducted based on predefined criteria. Consecutively, relatedness to the study interventions and potential impact on the participant will be determined. A medical intervention will be applied if needed. The adverse event will be documented thoroughly, including details such as the nature of the event, severity, interventions undertaken, and any outcomes. The adverse event information will be communicated promptly to relevant study personnel, including the PI (MK), study coordinators, and site investigators. Ongoing medical follow-up for the participant experiencing the adverse event will be ensured. Resolution or stabilization of the adverse event will be monitored, and the management plan will be adjusted accordingly.

If the adverse event significantly impacts the participant’s safety or ability to continue, withdrawal from the study will be considered. The withdrawal decision, reasons, and necessary follow-up care will be documented.

## Frequency and plans for auditing trial conduct {23}

The Trial Steering Committee will meet quarterly to provide overarching supervision of the trial, including ensuring that the trial is conducted according to the protocol, ethical guidelines, and regulatory requirements.

The Project Management Group will meet monthly to review trial conduct, including participant recruitment, adherence to the protocol, data collection processes, and any adverse events. These meetings will ensure ongoing oversight and timely identification of any issues that need to be addressed.

Given that this study involves a relatively low-risk intervention, the establishment of an independent data monitoring committee was not considered. However, an internal Data Monitoring Committee will be developed and meet quarterly. Also, to ensure rigorous monitoring, one person from the research team (PR) will be responsible for auditing the trial quarterly in both settings and, therefore, will not be directly involved in the primary data collection. The person will be responsible for safety monitoring and assessing trial conduct and any emerging safety data.

## Plans for communicating important protocol amendments to relevant parties (e.g., trial participants, ethical committees) {25}

Important protocol amendments, such as changes to eligibility criteria, outcomes, or analyses, are permitted only by the PI (MK). The parties that will need to be communicated in case of essential protocol modifications include all co-investigators (KE, MS, PŚ, MB, AK, PR), personnel at each study site involved in the day-to-day conduct of the trial, and the Bioethics Committee at the Kuyavian-Pomeranian District Medical Chamber in Torun, Poland. Any changes will be appropriately updated in the clinical trial registry (ClinicalTrials.gov). They will also be reported along with the trial results.

## Dissemination plans {31a}

The dissemination plans include presenting the study results at relevant scientific conferences and meetings attended by healthcare professionals and submitting manuscripts reporting the results to peer-reviewed journals for publication.

## Discussion

The main findings of the planned multicenter randomized clinical trial will be the effect of combined usage of povidone-iodine irrigation and vancomycin powder in comparison to the use of povidone-iodine irrigation alone on the PJI rate in patients undergoing primary THA and TKA.

PJI is a severe complication of TKA and THA, leading to substantial morbidity, increased healthcare costs, and often necessitating revision surgeries [23]. Investigating the efficacy of an intervention aimed at reducing PJI rates addresses a clinically relevant issue, potentially improving patient outcomes and reducing the burden on healthcare systems. Despite various infection prevention measures, the incidence of PJI remains a significant concern [24]. Therefore, there is a need to explore additional interventions that may further reduce the risk of PJI.

Study protocols serve as the foundation for transparent, replicable research practices that strengthen the credibility of scientific investigations. Mandatory pre-registration of research protocols and their publication is another crucial step toward improving research standards in orthopedics [21]. When reporting the content of a protocol for clinical trial purposes, the Standard Protocol Items: Recommendations for Interventional Trials (SPIRIT) Checklist constitutes a suggested tool [25]. SPIRIT 2013 Statement provides evidence-based recommendations for the minimum content of a clinical trial protocol [26].

The multicenter design of the planned RCT should be highlighted. A multicenter approach facilitates the recruitment of a larger and more diverse patient cohort. This increased sample size improves statistical power, enabling more robust analyses and more reliable conclusions regarding the comparative efficacy of the interventions. Involving multiple centers increases the diversity of the patient population, enhancing the generalizability of study findings. It also helps mitigate the impact of center-specific biases. Findings from a multicenter trial are more likely to apply to a broader patient population and various clinical settings. This enhances the external validity of the study, making the results more relevant and applicable to real-world scenarios. Collaborating with multiple centers can expedite recruitment, as the study can tap into a larger pool of potential participants, which is particularly important for trials focusing on relatively infrequent outcomes, such as PJIs.

In this study protocol comparing the effect of combined povidone-iodine irrigation and topical vancomycin powder to povidone-iodine irrigation alone on the incidence of PJI in patients undergoing primary TKA or THA, we have opted not to perform an Intention-to-Treat (ITT) analysis. This decision is based on several critical considerations. First, the effectiveness of the interventions is highly dependent on strict adherence to the treatment protocol, making the Per-Protocol (PP) analysis more suitable for assessing the true efficacy of the treatments under ideal conditions. Second, by focusing on patients who adhered strictly to the assigned intervention, we aim to minimize the biases introduced by protocol deviations, non-adherence, or crossover, which are particularly impactful in surgical and procedural trials.

## Trial status

Protocol version number ClinicalTrials.gov Identifier: NCT05972603 of 2 August 2023, https://classic.clinicaltrials.gov/ct2/show/NCT05972603. Recruitment started on 1 July 2022. Current recruitment status: recruiting. Estimated study completion date: December 2024.

## Data Availability

During the trial, only the PI (MK), two study coordinators (KE and MB), and one co-investigator dedicated to auditing the trial (PR) will have access to the trial dataset, including the personal data of participants and their intervention assignment. Also, if needed, the Bioethics Committee at the Kuyavian-Pomeranian District Medical Chamber in Torun, Poland, will have access to the dataset to monitor the study’s conduct and ensure compliance with ethical standards. The other co-investigators (PŚ, MS), involving the person responsible for statistical analysis (AK), will have access to the trial dataset in an anonymized form and without information concerning which group was assigned to which intervention. After finalizing the trial, the anonymized dataset will be available in a public, open-access repository. The needed details on how to access the data, like the name of the repository, will be reported along with reporting of the trial results.

## Abbreviations

ASA: American Society of Anesthesiologists
MSIS: Musculoskeletal Infection Society
PI: Principal Investigator
PJI: Periprosthetic joint infection
THA: Total hip arthroplasty
TKA: Total knee arthroplasty
